# DNA Damage and Its Cellular Response in Mother and Fetus Exposed to Hyperglycemic Environment

**DOI:** 10.1155/2014/676758

**Published:** 2014-08-14

**Authors:** Jusciele Brogin Moreli, Janine Hertzog Santos, Clarissa Ribeiro Rocha, Débora Cristina Damasceno, Glilciane Morceli, Marilza Vieira Rudge, Estela Bevilacqua, Iracema Mattos Paranhos Calderon

**Affiliations:** ^1^Graduate Program in Gynecology, Obstetrics and Mastology, Botucatu Medical School, São Paulo State University (UNESP), SP, Brazil; ^2^Laboratory of Molecular Carcinogenesis, National Institute of Environmental Health Sciences (NIEHS), NC, USA; ^3^Department of Microbiology, Institute of Biomedical Sciences, University of São Paulo (USP), SP, Brazil; ^4^Department of Cell and Developmental Biology, Institute of Biomedical Sciences, USP, University of São Paulo, São Paulo, Brazil; ^5^Department of Obstetrics and Gynecology, Botucatu Medical School, São Paulo State University (UNESP), Distrito de Rubião Jr. s/n, 18618-000 Botucatu, SP, Brazil

## Abstract

The increased production of reactive oxygen species (ROS) plays a key role in pathogenesis of diabetic complications. ROS are generated by exogenous and endogenous factors such as during hyperglycemia. When ROS production exceeds the detoxification and scavenging capacity of the cell, oxidative stress ensues. Oxidative stress induces DNA damage and when DNA damage exceeds the cellular capacity to repair it, the accumulation of errors can overwhelm the cell resulting in cell death or fixation of genome mutations that can be transmitted to future cell generations. These mutations can lead to and/or play a role in cancer development. This review aims at (i) understanding the types and consequences of DNA damage during hyperglycemic pregnancy; (ii) identifying the biological role of DNA repair during pregnancy, and (iii) proposing clinical interventions to maintain genome integrity. While hyperglycemia can damage the maternal genetic material, the impact of hyperglycemia on fetal cells is still unclear. DNA repair mechanisms may be important to prevent the deleterious effects of hyperglycemia both in mother and in fetus DNA and, as such, prevent the development of diseases in adulthood. Hence, in clinical practice, maternal glycemic control may represent an important point of intervention to prevent the deleterious effects of maternal hyperglycemia to DNA.

## 1. Introduction

**Figure 1:**
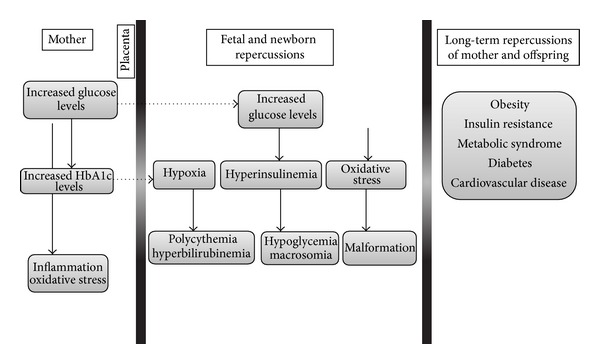
Schematic representation of outcomes classically associated with hyperglycemic pregnancies. The representation does not show all possible relationships between the characteristics that are depicted. Adapted from Metzger et al. [75], Negrato et al. [11], and Fraser and Lawlor [52].

Diabetes mellitus (DM) is a metabolic disease characterized by hyperglycemia resulting from a defect in insulin action and/or production [1]. In pregnancy, hyperglycemia poses a risk to maternal, fetal, and perinatal health [2–4]. Perinatal complications of a diabetic pregnancy include malformations, macrosomia, hypoxia, hypoglycemia, cardiomyopathy, hyperbilirubinemia, and hyperinsulinemia [3, 5–9]. The current literature acknowledges this adverse environment as associated with increased long-term risk for the development of diabetes, obesity, cardiovascular, and malignant diseases (Figure 1) [9–14].

Previous findings by our group have shown that maternal hyperglycemia is also adversely involved in fetal development by changing the placental production of proinflammatory cytokines, that is, TNF-*α* (tumor necrosis factor alpha) [15, 16]. The cellular redox status may be an important connection between inflammation and adverse perinatal outcomes in hyperglycemic pregnancies [17]. There is considerable evidence that hyperglycemia and inflammation results in the generation of reactive oxygen species (ROS), ultimately leading to increased oxidative stress. In the absence of an appropriate antioxidant response, the system becomes overwhelmed leading to production of reactive molecules that can cause cellular damage and are responsible for the late complications of diabetes [17, 18]. During pregnancy the placenta is an additional source of ROS generation, contributing to oxidative stress even in normal pregnancies. This is increased in pregnancies complicated by preeclampsia, intrauterine growth restriction, and pregestational diabetes where oxidative and nitrative stress have been clearly documented [19, 20].

**Figure 2:**
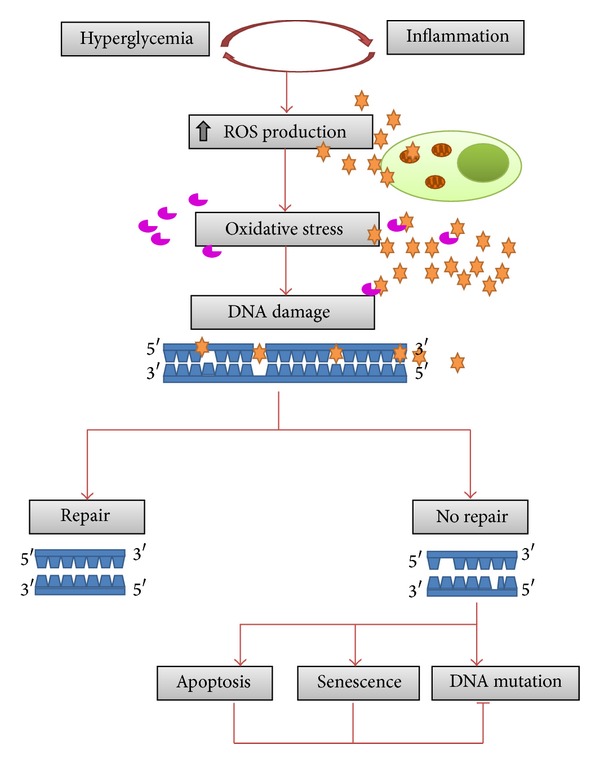
Hyperglycemia and inflammation are able to increased ROS production. When ROS production exceeds the detoxification and scavenging capacity of the cell, oxidative stress ensues. Oxidative stress induces DNA damage and when DNA damage exceeds the cellular capacity to repair it, the accumulation of errors can overwhelm the cell resulting in apoptosis, cell senescence, or fixation of genome mutations that will be transmitted to future cell generations. These mutations can lead to and/or play a role in cancer development.

Oxidative stress induces protein oxidation, lipid peroxidation, and DNA damage both in mitochondrial and nuclear DNA. Degradation processes can remove lipids and proteins but not DNA, which needs conversely to be repaired. When DNA damage exceeds the cellular capacity to repair it, the accumulation of errors can overwhelm the cell and result in cell death or the incorporation of genome mutations that can be transmitted to future cell generations if they occur in germ cells (Figure 2). In addition, mutations in somatic cells can promote genome instability and directly lead to various human diseases including cancer, neurological abnormalities, immunodeficiency, and premature aging [21–25].

Considering that hyperglycemia may alter genomic integrity and the consequences of this relationship to maternal and fetus genome is unclear, this review aims at (i) assessing the types and consequences of DNA damage during hyperglycemic pregnancy and lifelong risks, (ii) identifying the biological role of DNA repair during pregnancy, and (iii) proposing clinical interventions to maintain genome integrity.

## 2. Hyperglycemia-Induced Oxidative Stress and Its Effects on DNA Structure 

Hyperglycemia causes many of the major complications of diabetes including nephropathy, retinopathy, neuropathy, and macro- and microvascular damage [1]. To date, there is emerging evidence that oxidative stress significantly contributes to the progression of diabetes and its complications and induces alterations in embryonic and fetal development during pregnancy [18, 26]. Li and collaborators [27] found that mothers with GDM and their newborns had higher levels of 8-Isoprostaglandin F2*α* (an oxidative stress marker) than control group. Hyperglycemia induces ROS production during such processes as nonenzymatic glycosylation, increased generation of superoxide anion radical by the mitochondrial respiratory chain and the overactivation of NADPH oxidase (nicotinamide adenine dinucleotide phosphate-oxidase) [28, 29].

Overproduction of ROS is capable of altering the structure and function of all types of molecules including proteins, membrane lipids, and nucleic acids with serious consequences to cell viability [21, 30]. Different degradation processes can remove oxidized lipids and proteins. DNA, however, has to be repaired or in the case of mitochondrial DNA may even be removed. The latter is intrinsic to the various copies of mitochondrial genome present in each mitochondrion and the fact that many mitochondria populate a cell [21, 31]. ROS are able to induce DNA lesions as abasic sites (AP sites), single strand breaks, and double strand breaks and oxidize DNA bases. All four bases are susceptible to oxidative damage by ROS. However, due to the lower redox potential of guanine this base is more susceptible to oxidation [23, 30, 32]. The oxidized guanine (8-oxodG) has great biological importance as this is a mutagenic lesion that induces G-T transversions. It may also impair DNA replication and transcription and may be an intermediate for other types of lesions in DNA [23, 33].

Substantial evidence suggests that mitochondrial DNA may be more vulnerable than nuclear DNA to certain kinds of damage, in particular, ROS-mediated lesions [31, 34, 35]. Several reasons may underline this affirmation, including the immediate proximity of mitochondrial DNA to the electron transport chain in the inner mitochondrial membrane, which is the main source of endogenous ROS production. In addition, the repair of mitochondrial DNA lesions occurs only via base excision repair and unlike the nuclear genome, the mitochondrial DNA is not protected by histones [31, 34, 35].

It is important to remember that the genomes of all organisms are constantly being modified by reactive molecules that are produced endogenously, primarily via mitochondrial respiration or by environmental/exogenous physical, chemical, and biological agents including ultraviolet light, ionizing radiation, heavy metals, air pollutants, chemotherapeutic drugs, and inflammatory responses [25, 36].

## 3. Hyperglycemia, DNA Damage, and Pregnancy: Results of Experimental and Clinical Studies

In nonpregnant context, the relationship between type 1 diabetes mellitus (T1DM), type 2 diabetes mellitus (T2DM), and DNA damage is well established [42–48]. Little is known about DNA damage in pregnancy, especially in pregnancy complicated by pregestational (T1DM or T2DM) or gestational diabetes mellitus (GDM) [7, 37–39, 41, 49].

Experimental studies conducted in our laboratory with streptozotocin-induced diabetic rats showed that the levels of basal DNA damage in leukocyte of mothers with severe diabetes (blood glucose ≥ 300 mg/dL) and their respective fetus was higher when compared with the control group [37, 38]. Subsequently, Lima et al. [7] demonstrated that rats with severe diabetes and their offspring showed higher oxidatively generated DNA damage in leukocyte detected by Fpg (formamidopyrimidine-DNA glycosylase) and endonuclease III-sensitive sites when compared to mild diabetes group (blood glucose levels between 120 and 299 mg/dL). Taken together, these experimental results suggest that the intensity of diabetes is related to the levels of oxidative DNA damage. Thus, hyperglycemia may have repercussions at the DNA level that go beyond the pregnant mother.

In a pilot study, Qiu and collaborators [39] evaluated, in early pregnancy, levels of urinary 8-oxodG trying to determine an association with the risk of GDM development. They observed that the risk for GDM was higher in overweight women with urine 8-oxodG concentrations ≥8.01 ng/creatinine mg (OR = 5.36; 95% Cl 1.33–21.55) when compared with lean women who had 8-oxodG concentrations <8.01 ng/creatinine mg. Interestingly, levels of 8-oxodG in umbilical vein plasma in pregestational and control groups were reported to be similar [40].

Evaluation of telomere length is another way to estimate the stability of the genetic material. Telomeric length and telomerase activity (a reverse transcriptase that limits telomere attrition) were studied in mononuclear cells isolated from umbilical cord blood of pregnant women with pregestational diabetes (T1DM and T2DM) and GDM. No difference was found in cord blood telomere length in pregnancies of women with diabetes compared with control subjects, but higher telomerase activity was observed in Type 1 and GDM groups. The upregulation of telomerase may be a compensatory response to* in utero* oxidatively generated DNA and telomere damage [41].

Previous study demonstrated that telomerase is found in nuclei and mitochondria. Telomerase is able to decrease mitochondrial levels of ROS, especially in mitochondria [34, 50]. Recently, Li and collaborators [27] evaluated the mitochondrial translocation of human telomerase reverse transcriptase (hTERT) in mononuclear cells isolated from umbilical cord blood during pregnancies complicated by GDM with confirmed oxidative stress. They found that the ratio of mitochondrial/nuclei hTERT was increased significantly in the newborn of GDM mothers, suggesting that mitochondrial hTERT in cord blood mononuclear cells may have a protective effect on neonatal mitochondrial DNA in GDM pregnancies. The authors concluded that this dynamic translocation could be an* in utero* adaptive response of a fetus that is suffering from elevated oxidative stress and could help our understanding of the roles of oxidative stress in fetal programming.

A few years ago, epigenetic processes have been suggested as a link between maternal pregnancy diabetes and long-term offspring outcomes. Epigenetic modifications, such as DNA methylation, regulate gene expression without altering the DNA sequence. These alterations occur in response to environmental stimuli [51–54]. Recent studies compared the levels of global methylation in the placenta and umbilical cord blood among women with and without gestational diabetes, preeclampsia, and obesity. They found that the mother's metabolic problems during pregnancy may influence the epigenome in the offspring [51]. del Rosario et al. [54] found that epigenetic changes (DNA methylation) may increase the risk of type 2 diabetes; studies support this association but research in this area is still inconclusive [52].

In summary, the results found in the literature indicate that maternal and fetal cells, especially mononuclear cells of blood, respond differently to the hyperglycemic environment (Table 1). While it is clear that hyperglycemia can damage the maternal genetic material, the results in umbilical cord blood (fetal cells) remain unclear. It seems that umbilical cord blood cells have more efficient mechanisms working to protect the genome. Future investigations on the mechanisms involved in genome protection in fetal cells as well as the role of epigenetic changes may shed new light on the outcome on offspring born from women with gestational diabetes.

## 4. DNA Repair Mechanisms Are Important to Maintain the Genetic Stability

To maintain genetic stability organisms possess cellular mechanisms collectively termed the DNA damage response (DDR) to detect DNA lesions and signal their presence and promote their repair. Cells with DDR defects display higher sensitivity toward DNA damaging agents and many such defects lead to mutagenesis, cytotoxicity, cell death, and disease. In fact, genomic instability and defects in DDR are known to play a role in disease processes such as carcinogenesis, neurodegenerative disorders, immune deficiencies, infertility, aging, cardiovascular disease, and metabolic syndrome [30, 55]. In this session we will focus on DNA repair.

To repair different types of DNA lesions the cell counts on a variety of proteins that presumably undergo crosstalk to form a network for protection of the cellular genome. [25, 56–59].

Nucleotide excision repair (NER), mismatch repair (MMR), and base excision repair (BER) have been implicated in the repair of ROS-induced lesions in DNA. However, BER is the main mechanism involved in the removal of these lesions in nuclear DNA and is the unique mechanism demonstrated for mitochondria damaged DNA [31, 34, 35, 60]. BER predominantly repairs oxidized bases, AP sites, and single strand breaks. In general, BER initiates with the action of a DNA glycosylase that is able to remove the damaged base resulting in an AP site. The AP site is then cleaved by the AP-endonuclease, allowing the DNA polymerase (*β* in the nucleus or gamma in the mitochondria) to synthesize the repair patch. The latter is relegated based on DNA ligase III activity [60].

## 5. The Possible Role of DNA Repair during Pregnancy and Diabetes Disease

Studies have demonstrated the importance of DNA repair genes in pregnancy and perinatal development. Patients with mutations in XPD (Xeroderma pigmentosum D) and GTF2H5 (general transcription factor IIH, polypeptide 5), genes involved in the NER pathway and in transcription-couple repair, have the DNA repair diseases: trichothiodystrophy (TTD), xeroderma pigmentosum (XP), Cockayne syndrome (CS), cerebro-ocular facial syndrome (COFS), or a combination [24, 61, 62]. The pregnancies in which the fetus had TTD were at significantly increased risk of preeclampsia, HELLP (hemolysis, elevated liver enzymes, and low platelet count) syndrome, and elevated mid-trimester maternal serum human chorionic gonadotropin levels. The affected fetus had decreased fetal movement and preterm delivery with higher index of small for gestational age fetus [63]. The authors hypothesized that mutations observed in TTD patients affect placental development. Two years later, the same group revealed that only a specific subset of XPD mutations, which lead to TTD but are unrelated to XP, results in higher risk to develop preeclampsia and other gestational complications [64]. A functional polymorphism (199 Arg-399Gln) in XRCC1 (X-ray repair complementing defective repair in Chinese hamster cells 1), a gene involved in the BER pathway, showed higher frequency among patients with preeclampsia (OR 1.65; 95% CI 1.23–2.19) in an Iranian population [65]. However, this polymorphism was not associated with T2DM in a Polish population [66].

DNA repair was evaluated in lymphocytes of nonpregnant patients with T1DM and T2DM [45, 46]. The results of Blasiak et al. [45] suggest that T2DM may be associated not only with elevated levels of oxidative DNA damage but also with decreased efficacy of DNA repair. In an elegant study Pácal et al. [46] compared DNA damage and repair in lymphocytes of T1DM children, T1DM adults, and T2DM adults. The T2DM diabetics exhibited a significant increase in DNA damage and decreased DNA repair capacity when compared with T1DM (both children and adults). T1DM children displayed a significant decrease of DNA damage and increase in DNA repair when compared with diabetic adults (both T2DM and T1DM). These findings indicate significant age- and DM type-related changes of DNA damage and repair capacity in diabetic subjects.

In summary, the data available suggest that DNA repair mechanisms are involved in the long-term consequences of diabetes in T1DM and T2DM subjects. In pregnancy, DNA repair genes may affect the harmony of maternal-fetal interface resulting in adverse perinatal results.

## 6. Diabetes and Cancer

Epidemiologic evidence suggests that diabetic patients are at significantly higher risk for many types of cancer. T2DM, GDM, and cancer share many risks factors but potential biological links between the two diseases are unclear [67, 68]. Meta-analyses have reported an increased risk of liver, pancreatic, renal, endometrial, colorectal, bladder, and breast cancer as well as an increase in the incidence of non-Hodgkin lymphoma in T2DM subjects [68]. For those with T2DM compared with those without diabetes, the greatest increase in risk is for hepatocellular carcinoma (RR 2.5; 95% CI 1.8–3.5), with the relative risk for cancer at other sites being between 1.18 (95% CI 1.05–1.32) for breast cancer and 2.22 (95% CI 1.8–2.74) for endometrial cancer in those with diabetes [68, 69]. A prospective cohort study with 37.926 women in Jerusalem observed no cases of pancreatic cancer in the women with T1DM; however, women with a history of GDM showed a relative risk of pancreatic cancer of 7.1 (95% confidence interval 2.8–18.0) [70]. Similar results were observed with a late cohort in Israel [71]. In addition to the relationship between GDM and pancreatic cancer, the authors observed an increased risk of hematologic malignancies like non-Hodgkin's lymphoma, Hodgkin's lymphoma, and acute myeloid leukemia in the same population [71]. A relationship between GDM and breast cancer was found in a New Zealand population, but when studying the U.S. population this association was not observed [72, 73].

Experts assembled jointly by the American Diabetes Association (ADA) and the American Cancer Society (ACS) reviewed the possible biological links between diabetes and cancer risk. They suggested that diabetes may influence the neoplastic process by several mechanisms, including hyperinsulinemia, hyperglycemia, or chronic inflammation without reference to DNA damage and repair [67]. However, the increase in DNA damage and decrease in DNA repair observed in T2DM subjects may provide a new link between diabetes and cancer [45, 60, 74].

## 7. Proposed Clinical Intervention Strategy for Maintenance of Genomic Integrity

### 7.1. Control of Maternal Hyperglycemia

Maternal hyperglycemia is able to induce fetal hyperglycemia [1, 4] (Figure 1), increase the release of proinflammatory cytokines [15, 16], and ROS production [17, 18] (Figure 2). Thus, it appears that maternal glycemic control during hyperglycemic pregnancies is an old and safe strategy to assure maintenance of genomic integrity. Clinical studies have already demonstrated the benefits of maternal glycemic control during pregnancy and how to maintain optimal glucose levels without gestational risk [75, 76].

Nonpregnant adults with diabetes and pregnant women with GDM or pregestational diabetes (T1DM or T2DM) presented different glycemic recommendations [1]. During pregnancy, the glycemic limits are stricter than in nonpregnant state to prevent alteration in both maternal and fetal health [1, 75, 76]. Based on recommendations from the Fifth International Workshop-Conference on Gestational Diabetes Mellitus [77] and ADA's statement [1] it is important to maintain maternal capillary glucose concentrations at <95 mg/dL (<5.3 mmol/L) in the fasting state, <140 mg/dL (<7.8 mmol/L) at 1 h, and <120 mg/dL (<6.7 mmol/L) 2 h after starting the meal. For women with overt diabetes who become pregnant, the optimal glycemic goals are premeal, bedtime, and overnight glucose between 60 and 99 mg/dL (3.3–5.4 mmol/L) and peak postprandial glucose between 100 and 129 mg/dL (5.4–7.1 mmol/L) and HbA1C of 6.0% [78].

Diet therapy, control of weight gain, and increasing physical activity are the standard treatment of GDM [77]. Insulin administration is only performed for pregnant women who fail to maintain glycemic goals as well as to the ones who show signs of excessive fetal growth or overt diabetes. It is recommended that insulin administration be individualized to achieve the glycemic goals stated [77]. During the last decade, there was an increased interest in the use of oral antihyperglycemic agents as an alternative to insulin in achieving good glycemic control. However, the results are inconclusive [79, 80].

### 7.2. Antioxidant Supplementation during Pregnancy

Antioxidant supplementation is a questionable strategy during pregnancy. The effects of vitamin C supplementation, alone or in combination with other supplements, have been evaluated on pregnancy outcomes. No difference was seen in the risk of stillbirth, perinatal death, birth weight, or intrauterine growth restriction between women supplemented with vitamin C alone or in combination with other supplements and placebo. In fact, women supplemented with vitamin C alone or combined with other supplements were at increased risk of giving preterm birth [81]. The same researchers also determined the effectiveness and safety of any vitamin supplementation on the risk of spontaneous miscarriage, maternal adverse outcomes, and fetal and infant adverse outcomes. The results indicated that vitamin supplements, alone or in combination, prior to pregnancy or in early pregnancy, did not prevent miscarriage or stillbirth. However, it was found that women taking vitamin supplements were less likely to develop preeclampsia while more likely to have multiple pregnancies [82, 83]. Mothers that have taken antioxidant supplementation during pregnancy had decreased frequency of micronucleus (a test used to quantify chromosomal damage) in peripheral blood mononuclear cells prior to and after hydrogen peroxide exposure. The additional antioxidants intake during pregnancy was also beneficial to reduce the frequency of micronucleus after hydrogen peroxide exposure in cord blood cells. The data demonstrated a positive effect of antioxidant supplementation on micronucleus frequency [84]. Experimental results in a model of diabetic pregnancy indicate that high doses of dietary antioxidants were need to normalize the development of offspring. However, treatment with such high doses may also have adverse effects in nondiabetic pregnancy [85].

It is clear based on the above findings that results about antioxidant supplementation during pregnancy are still inconclusive, and little is known about their impact at the DNA level. Despite this fact, taken together the data support the notion that maternal glycemic control is a good and safe plan to reduce the factors associated to genomic instability in hyperglycemic pregnancy.

## 8. Conclusions

Although it is clear that hyperglycemia can damage the maternal genetic material, the results obtained for cord blood are not yet clear. The data seem to support the hypothesis that umbilical cord blood cells have more efficient mechanisms to protect the genome than the mother's cells. DNA repair may be thus considered an important mechanism to prevent the deleterious effects of hyperglycemia in the genetic material. However, functional studies demonstrating the ability of DNA repair mechanisms in dealing with insults resulting from hyperglycemia during pregnancy need to be developed. For the time being, the control of maternal hyperglycemia seems a safe and important strategy to prevent the deleterious effects of hyperglycemia on maternal and potentially fetal DNA.

## Figures and Tables

**Table 1:** Maternal and fetal DNA integrity in hyperglycemic environment.

Reference	Study type	Type of diabetes	Sample	Evaluation	Main results
[37]	Experimental	Severe	Maternal leukocytes	Comet assay	Basal DNA damage in severe diabetes
[38]	Experimental	Severe	Fetal leukocytes	Comet assay	Basal DNA damage in severe diabetes
[7]	Experimental	Mild and severe	Maternal and fetal leukocytes	Comet Assay with Fpg and Endo III enzymes∗	Oxidative DNA damage in severe diabetes
[39]	Clinical	GDM	Maternal urine	8-oxodG levels	Elevated in early pregnancy that results in GDM
[40]	Clinical	Pregestational	Umbilical vein plasma	8-oxodG levels	No difference
[41]	Clinical	Pregestational and GDM	Cord blood mononuclear cell	Telomere length and telomerase activity	Telomerase activity higher in cord blood from T1DM and GDM
[27]	Clinical	GDM	Cord blood mononuclear cells	Mitochondrial translocation of hTERT	Increased mitochondrial hTERT levels in GDM

GDM: gestational diabetes mellitus; hTERT: human telomerase reverse transcriptase. ∗The endonuclease III (Endo III) and formamidopyrimidine-DNA glycosylase (FPG) are enzymes used to detect oxidative DNA damage.
